# Regulation of virulence and β-lactamase gene expression in *Staphylococcus aureus* isolates: cooperation of two-component systems in bloodstream superbugs

**DOI:** 10.1186/s12866-021-02257-4

**Published:** 2021-06-25

**Authors:** Sanaz Dehbashi, Hamed Tahmasebi, Behrouz Zeyni, Mohammad Reza Arabestani

**Affiliations:** 1 Microbiology Department, Faculty of Medicine, Hamadan University of Medical Sciences, Hamadan, Iran; 2 School of Medicine, Shahroud University of Medical Sciences, Shahroud, Iran; 3 Nutrition health Research center, Hamadan University of Medical Sciences, Hamadan, Iran

**Keywords:** Methicillin-resistant *Staphylococcus aureus*, Virulence factors, β-Lactamase, Antibiotic resistance

## Abstract

**Background:**

Methicillin-resistant *Staphylococcus aureus* (MRSA)-bloodstream infections (BSI) are predominantly seen in the hospital or healthcare-associated host. Nevertheless, the interactions of virulence factor (VFs) regulators and β-lactam resistance in MRSA-BSI are unclear. This study aims to characterize the molecular relationship of two-component systems of VFs and the expression of the β-lactamase gene in MRSA-BSI isolates. In this study, 639 samples were collected from BSI and identified by phenotypic methods. We performed extensive molecular characterization, including *SCCmec* type, *agr* type, VFs gene profiles determinations, and MLST on isolates. Also, a quantitative real-time PCR (q-RT PCR) assay was developed for identifying the gene expressions.

**Results:**

Ninety-one (91) *S. aureus* and 61 MRSA (67.0%) strains were detected in BSI samples. The presence of VFs and *SCCmec* genes in MRSA isolates were as follows: *tst* (31.4%), *etA* (18.0%), *etB* (8.19%), *lukS-PVL* (31.4%), *lukF-PV* (18.0%), *lukE-lukD* (16.3%), *edin* (3.2%), *hla* (16.3%), *hlb* (18.0%), *hld* (14.7%), *hlg* (22.9%), *SCCmecI* (16.3%), *SCCmecII* (22.9%), *SCCmecIII* (36.0%), *SCCmecIV* (21.3%), and *SCCmecV* (16.3%). Quantitative real-time PCR showed overexpression of *mecRI* and *mecI* in the toxigenic isolates. Moreover, *RNAIII and sarA* genes were the highest expressions of MRSA strains. The multi-locus sequence typing data confirmed a high prevalence of CC5, CC8, and CC30. However, ST30, ST22, and ST5 were the most prevalent in the resistant and toxigenic strains.

**Conclusion:**

We demonstrated that although regulation of β-lactamase gene expressions is a significant contributor to resistance development, two-component systems also influence antibiotic resistance development in MRSA-BSI isolates. This indicates that resistant strains might have pathogenic potential. We also confirmed that some MLST types are more successful colonizers with a potential for MRSA-BSI.

**Supplementary Information:**

The online version contains supplementary material available at 10.1186/s12866-021-02257-4.

## Background

Bacteremia is defined as the presence of bacteria in the blood and may be detected by blood culture analysis for particular bacterial microbes [1, 2]. Traditionally methicillin-resistant *Staphylococcus aureus* (MRSA)- bloodstream infection (BSI) has been regarded as only a hospital-acquired infection (HAI) [3, 4]. Following the extensive use of a new antibiotic, a small number of MRSA successfully develop drug resistance. They develop mutations in the genome or resistant genes [2, 5]. Thus, new strains of bacteria resistant to existing antibiotics are developed and referred to as superbugs [6]. Multidrug resistance (MDR) was defined as non-susceptibility to at least one antibiotic in three or more antibiotic groups. Extensive drug resistance (XDR) was defined as non-susceptibility to at least one antibiotic in all except two or fewer groups, and pan drug-resistance (PDR) was defined as non-susceptibility to all antibiotics in all groups [6, 7].

The principal and most significant difference between methicillin-susceptible and MRSA is the gene cassette embedded in the chromosome, also known as staphylococcal chromosomal cassette mec (*SCCmec*) [8]. The main element of *SCCmec* is the *mecA* gene, which ensures that the host is resistant to known β-lactams [9]. *S. aureus* also expresses a wide array of virulence factors that provide survival during infection. The *agr* locus regulates more than 70 genes, including 23 virulence genes [10, 11]. It contains two divergent promoters, P2 and P3. The P2 promoter drives transcription of an auto-inducing signal transduction module composed of four genes, *agrBDCA* [10], which further activates transcription from the P2 and P3 promoters and completes the auto-inducing circuit [10, 12].

In addition to two-component systems, several global transcriptional regulators function in *S. aureus* virulence factors [12]. Nevertheless, several global regulatory systems have been identified in *S. aureus*, including the staphylococcal accessory regulator A (*sarA*). *SarA* has initially been thought to antagonize the effect of *agr* but later was found to have an additive effect. Mutations in either *agr* or *sarA* attenuate virulence; however, *agr-sarA* double mutant leads to the loss of virulence [10, 12, 13]. Moreover, the two-component system *SaeRS* is transcribed from a four-gene operon, further encoding the auxiliary genes *saeP* and *saeQ* [14].

While limited information is available regarding the risk for BSI among minority patients, there is no clear information regarding the associations of two-component systems and the expression of the β-lactamase gene in MRSA-BSI. Therefore, the primary aim of this investigation was to characterize and determine the risk factors for patients with BSI. A secondary purpose was to assess sequence types and clonal complex of superbugs in patients with MRSA-associated BSI.

## Results

Out of 639 BSI samples, 91 (14.2%) *S. aureus* was isolated. Also, 53 (58.2%) and 38 (41.7%) isolates were collected from female and male patients.

### Antibiotics susceptibility patterns

Antibiotic resistance pattern information is presented in Table 1. Out of 91 isolates of *S. aureus*, 83 (91.2%) isolates were sensitive to linezolid. Moreover, 74 (81.3%) and 61 (67.0%) isolates were resistant to penicillin and cefoxitin, respectively. Fifty (54.9%) isolates were resistant to at least seven antimicrobials and considered as MDR strain.

**Table 1:** Frequency of antibiotic resistance, virulence factors, and SCCmec types, in BSI collection of MRSA and MSSA strains

Antimicrobial agent	MRSA (*n* = 61)			*P*^*#*^	MSSA (*n* = 30)			*P*^*#*^
	R	I	S		R	I	S	
Cefoxitin	61	0	0	0.004	0	0	30	0.025
Gentamycin	38	8	16	0.03	1	0	29	0.012
Erythromycin	19	16	6	0.03	0	0	30	0.025
Amikacin	11	18	32	0.12	0	0	30	0.025
Ciprofloxacin	33	12	16	0.021	0	0	30	0.025
Clindamycin	19	13	29	0.022	0	0	30	0.025
Chloramphenicol	8	16	37	0.65	7	0	23	0.65
Linezolid	8	19	34	0.040	0	0	30	0.025
Daptomycin	11	0	50	0.79	0	0	30	0.025
Penicillin	61	0	0	0.001	13	9	8	0.059
Gatifloxacin	40	11	12	0.025	9	1	20	0.025
Trimethoprim/sulfamethoxazole	11	0	50	0.82	0	0	30	0.025
MDR	56			0.051	4			0.95
XDR	24			0.072	0			–
PDR	8			0.22	0			–
SCCmec types								
*SCCmecI*	10			0.019	0			–
*SCCmecII*	14			0.041	0			–
*SCCmecIII*	24			0.020	0			–
*SCCmecIV*	13			0.008	0			–
*SCCmecV*	10			0.045	0			–
Virulence factor genes								
*tst*	19			0.021	0			–
*etA*	11			0.001	0			–
*etB*	5			0.071	0			–
*lukS-PVL*	19			0.035	0			–
*lukF-PV*	11			0.040	0			–
*lukE-lukD*	10			0.055	0			–
*edinA*	2			0.037	0			–
*hla*	10			0.111	0			–
*hlb*	13			0.018	0			–
*hld*	9			0.068	0			–
*hlg*	14			0.018	0			–

*S* Susceptible, *R* Resistant, *I* Intermediate; ^#^Statistical relationship between chi-square test between different variables with significant level ≤ 0.05

### The prevalence of virulence and *SCCmec* genes

Based on Table 1, out of 91 isolates of *S. aureus*, 9 (15.7%) isolates carry all virulence and *SCCmec* genes and are considered superbug strains. Among 61 MRSA strains, *SCCmecIII* (36.3%) and *SCCmecIV* (21.3%) types were the most common. Moreover, the frequency of VFs genes in MRSA strains was as follows: *tst*, and *lukS-PVL* genes in 19 isolates (31.4%), *hlg* in 14 isolates (22.9%), and etA, *lukF-PV,* and *hlb* genes in 11 isolates (18.0%). None of the methicillin-susceptible *Staphylococcus aureus* (MSSA) strains carried the VF genes.

### Molecular analysis of β-lactamase and virulence regulatory genes

**Fig. 1:**
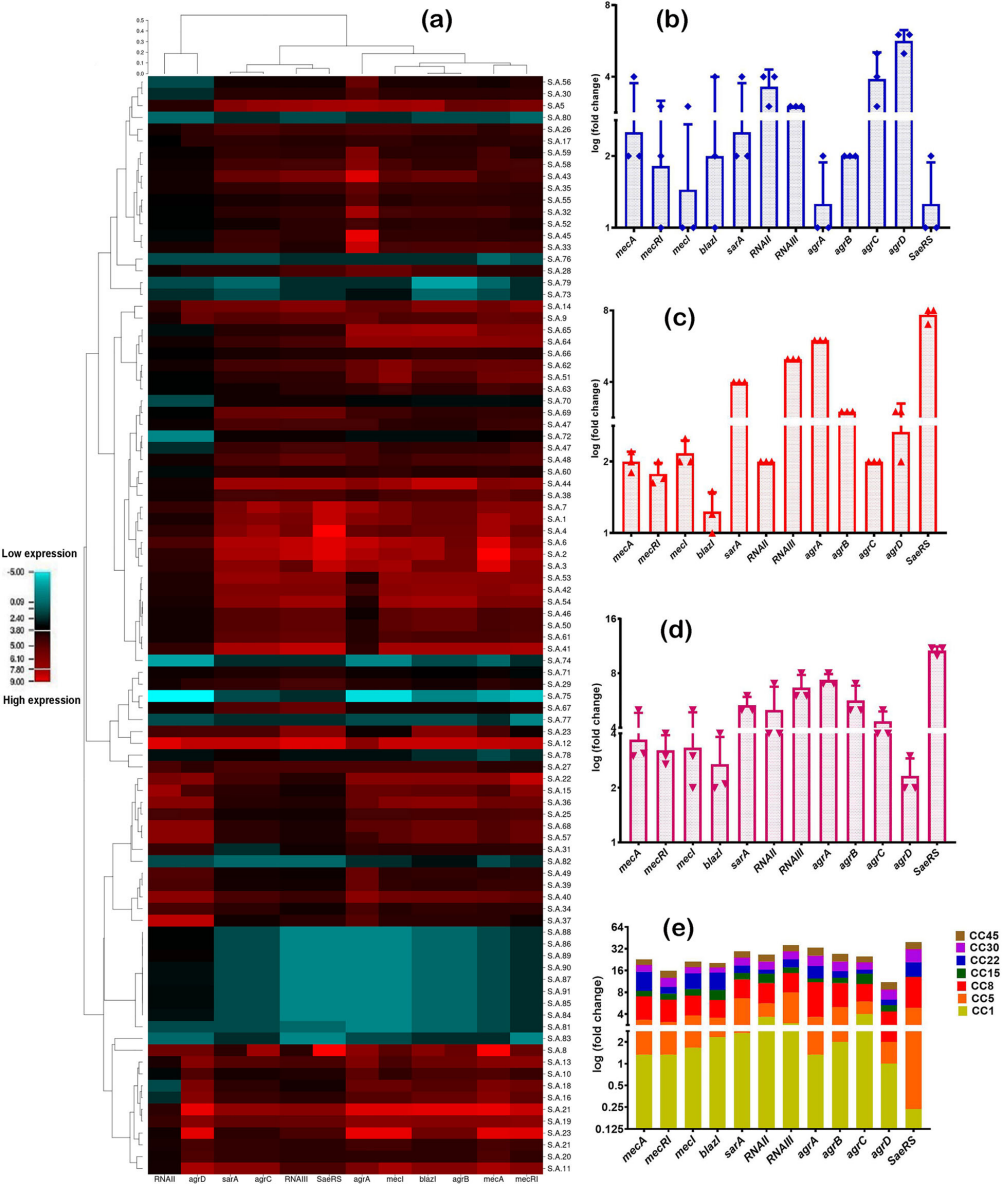
Differential expression of virulence and β-lactamase regulatory genes in BSI isolates of *S. aureus*. **a**: Heatmap of virulence and β-lactamase regulatory genes expression patterns in all 91 *S. aureus*. Red represents up-regulation and blue down-regulation relative to control. **b**: The expression levels of β-lactamase regulatory genes in MDR, XDR, and PDR strains, discriminated based on *p*-value and log_2_ (fold-change) at an α level of 0.05. **c**: The expression levels of β-lactamase regulatory genes in hemolysin producer, toxin producer, and non-virulent strains, discriminated based on *p*-value and log_2_ (fold-change) at an α level of 0.05. **d** The expression levels of virulence regulatory genes in MDR, XDR, and PDR strains, discriminated based on *p*-value and log_2_ (fold-change) at an α level of 0.05. **e**: The expression levels of virulence regulatory genes in hemolysin producer, toxin producer, and non-virulent strains, discriminated based on *p*-value and log_2_ (fold-change) at an α level of 0.05. Error bars standard errors: 0.05. Student’s t-test and Tow-Way ANOVA test were performed for testing differences between groups. *: *p* < 0.05, **: *p* < 0.001, ***: *p* < 0.0001

The results of the expression analysis of β-lactamase and virulence regulatory are shown in Fig. 1. Down-regulation for the *SaeRS*, *sarA,* and *RNAIII* genes was observed in antibiotic-sensitive isolates. Moreover, β-lactamase regulatory genes indicated the highest expression levels in virulent isolates. Interestingly, *agr* locus did not antagonize *mecA*, *mecI,* and *blaZ* genes in the MRSA strains. In total, these data suggest that *agr* locus acts as a regulatory bridge between antibiotic resistance and virulence factor production in *S. aureus*. Also, for the overexpression of *sarA*, *RNAIII, SaeRS* genes, the high expression was seen in superbug isolates.

### Analysis of hit-map tree of gene expression

The comparison of expression level for the virulence and β-lactamase regulatory genes in all 91 isolates of *S. aureus* is depicted in Fig. 1. The heat-map reflects the correlation between the virulence and β-lactamase regulatory genes in different strains. Compared to the control, a high-expressions of *agr* locus, *sarA* and *SaeRS* were found in MRSA and virulent strains. Also, in MRSA and MSSA strains, the *RNAII* and *RNAIII* genes had different expressen. Furtehr, the *mecRI,* and *mecI* regulatory genes were significantly more expressed in MRSA, toxigenic and hemolysin positive strains as compared to MSSA and non-virulent strains.

When comparing expression of *blaZI* and *mecA* genes to controls, the largest difference was found in the toxigenic and hemolysin positive strains, and the non-virulent strains. Indeed, most of the *agr* locus and *SaeRS* genes were expressed deficiently in non-toxigenic and MSSA strains. Also, *mecRI* and *mecI* regulatory genes are overexpressed in the toxigenic isolates.

### Analysis of MLST dendrogram phylogenic tree

**Fig. 2:**
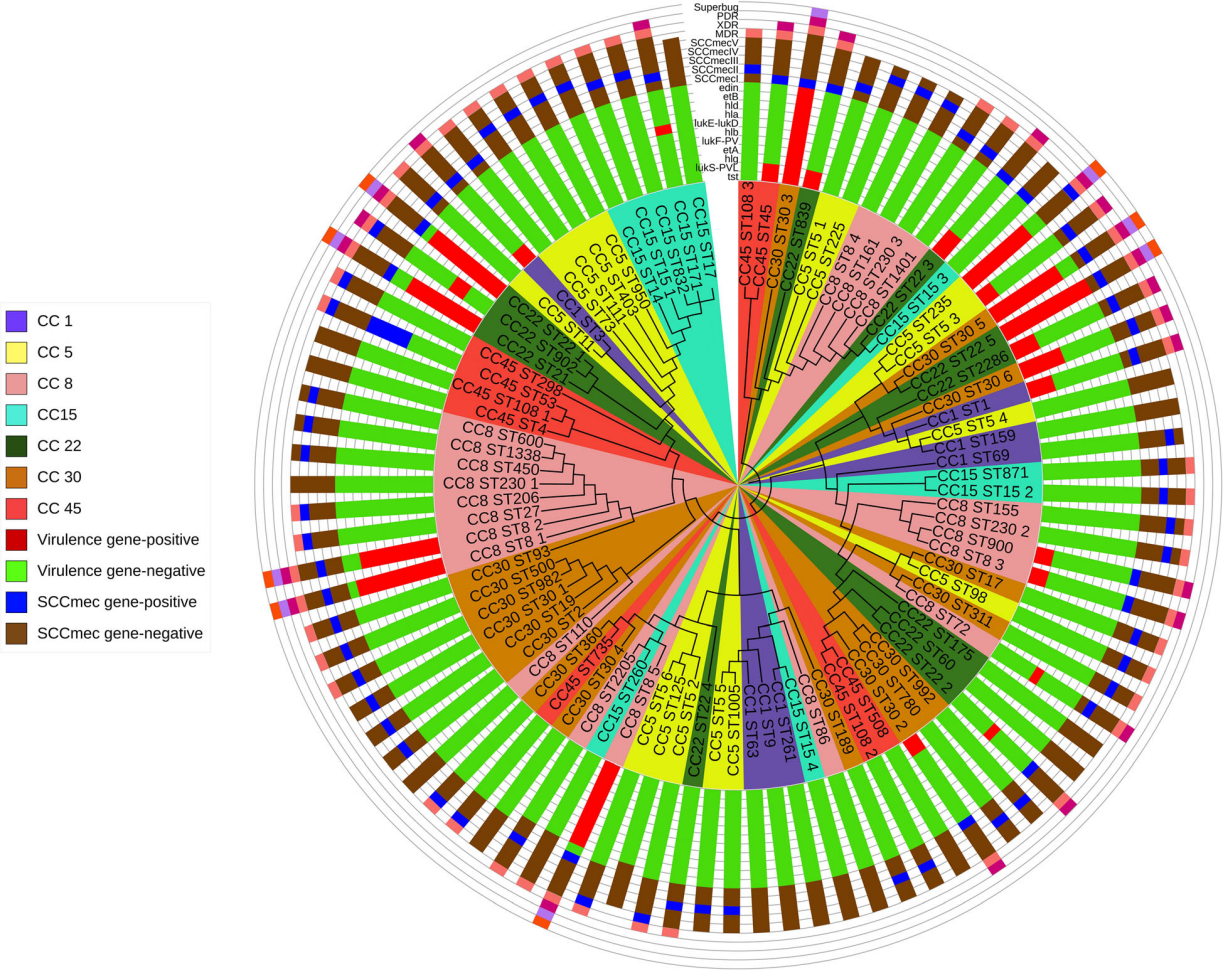
The multi-locus sequence typing (MLST) phylogenetic tree, unrooted, maximum likelihood, and circular-dendrogram clustering of *S. aureus* based on sequence type (ST) profiles. The circular-dendrogram was estimated by neighbor-joining using the k2 + G model, with MEGA version 6 and Figtree version 1.4.4

The neighbor-joining tree based on nucleotide difference in sequence data of each housekeeping gene was constructed, as shown in Fig. 2. Sequencing of housekeeping genes of all 91 representative isolates of *S. aureus* showed seven CC and 64 unique STs. However, the ST30, ST22, and ST8 were the most common STs in *S. aureus* isolates. These three STs also showed the highest frequency in MDR, XDR, and PDR strains. Also, most of the SCCmec types were found in five CC of *S. aureus*: CC5, CC8, CC22, CC30, and CC45.

### Relationship between VFs and antibiotic resistance

The results of the statical analysis showed in Table 2. However, the virulence profiles were significantly associated with the antibiotic resistance profile (*p* ≤ 0.05). No statistical association between the VFs prevalence and linezolid and amikacin resistance was detected. Surprisingly, a strong relationship was observed between expression levels of virulence and β-lactamase regulatory genes (*p* ≤ 0.001). Further, a high prevalence of *SCCmec* was significantly detected in toxigenic and hemolysin positive isolates (*p* ≤ 0.05).

**Table 2:** Relationship virulence factors, antibiotic resistance and virulence, and β-lactamase regulatory genes in BSI collection of *S. aureus*

Virulence factor genes	Fold change of virulence and β-lactamase regulatory genes							Antibiotic resistance profiles
	*mecRI*	*mecI*	*mecA*	*blaZI*	*RNAIII*	*sarA*	*RNAII*	
tst ***	< 0.001	< 0.001	< 0.001	< 0.001	< 0.001	< 0.001	< 0.001	0.034
*etA*	< 0.001	< 0.001	< 0.001	< 0.001	< 0.001	< 0.001	< 0.001	0.695
*etB*	< 0.001	< 0.001	> 0.001	< 0.001	< 0.001	> 0.001	< 0.001	0.074
*lukS-PVL*	< 0.001	< 0.001	< 0.001	< 0.001	< 0.001	< 0.001	< 0.001	0.007
*lukF-PV*	< 0.001	< 0.001	< 0.001	< 0.001	< 0.001	< 0.001	< 0.001	0.049
*lukE-lukD*	> 0.001	< 0.001	< 0.001	< 0.001	< 0.001	< 0.001	< 0.001	0.014
*edinA*	> 0.001	< 0.001	< 0.001	< 0.001	< 0.001	< 0.001	< 0.001	0.019
*Hla*	< 0.001	< 0.001	< 0.001	< 0.001	< 0.001	< 0.001	< 0.001	0.082
*Hlb*	< 0.001	< 0.001	> 0.001	< 0.001	< 0.001	> 0.001	< 0.001	0.060
*Hld*	< 0.001	< 0.001	< 0.001	< 0.001	< 0.001	< 0.001	< 0.001	0.002
*Hlg*	< 0.001	< 0.001	< 0.001	< 0.001	< 0.001	< 0.001	< 0.001	0.003
**SCCmec types**								
SCCmecI**	< 0.05	< 0.05	< 0.05	< 0.05	< 0.05	< 0.05	< 0.05	0.004
*SCCmecII*	< 0.05	< 0.05	< 0.05	< 0.05	< 0.05	< 0.05	< 0.05	0.049
*SCCmecIII*	< 0.05	< 0.05	< 0.05	< 0.05	< 0.05	< 0.05	< 0.05	0.052
*SCCmecIV*	< 0.05	< 0.05	< 0.05	< 0.05	< 0.05	< 0.05	< 0.05	0.040
*SCCmecV*	< 0.05	< 0.05	< 0.05	< 0.05	< 0.05	< 0.05	< 0.05	0.012
	< 0.05	< 0.05	< 0.05	< 0.05	< 0.05	< 0.05	< 0.05	0.053
**Antibiotic resistant strains**								
MDR*	< 0.05	< 0.05	< 0.05	< 0.05	< 0.05	< 0.05	< 0.05	0.009
XDR	< 0.05	< 0.05	< 0.05	< 0.05	< 0.05	< 0.05	< 0.05	0.051
PDR	< 0.05	< 0.05	< 0.05	< 0.05	< 0.05	< 0.05	< 0.05	0.069

Asterisks indicate significant differences in gene expression levels between (*, *P* < 0.05; **, *P* < 0.01; ***, *P* < 0.001)

## Discussion

The study was conducted on MRSA and MSSA strains to determine the prevalence of VFs, *SCCmec* cassette, and regulation of virulence and β-lactamase gene expression in BSI isolates. The result revealed that the overall prevalence of penicillin and gatifloxacin resistant strains in *S. aureus* isolates 81.3 and 53.8%, respectively. The linezolid (8.79%) and amikacin (12.0%) sensitive strains also were the most abundant. Similar findings were reported previously [8, 15].

In the current study, out of 61 MRSA strains, *SCCmecI* and *SCCmec*V, *SCCmecII*, *SCCmecIII,* and *SCCmecIV* genes were detected in 16.3, 22.9, 36.3, and 21.3% of isolates. This finding was also reported by some researchers from South Africa [16], Saudi Arabia [17], and Venezuela [18]. Surprisingly, none of the MSSA strains carried the VF genes. Previous studies have demonstrated a significant relationship between VFs and the SCCmec cassette. These findings are replicated in the current study. However, this effect appears to be strain-dependent, as this positive correlation between virulence and *SCCmec* is not observed in all MRSA strains [19, 20].

Likewise, different virulence factors related to MRSA clonal lineages are presented in a study performed by Hamada et al. and Bhowmik et al., suggesting that MRSA harboring SCCmec type IV produce significantly more biomass under static conditions than SCCmec type I-III. Nevertheless, better biofilm formers and a high prevalence of VFs correspond to SCCmec type I-III rather than SCCmec type IV when dynamic conditions are used [21, 22]. However, Kim et al. noticed an association between SCCmec type IV or V and virulence factors. The high prevalence of such staphylococcal cassettes promotes *S. aureus* pathogenicity. Thus allowing the bacteria to persist in the environment [23].

Based on gene expression results, the expression of VFs regulatory genes (*sarA*, *SaeRS,* and *RNAIII*) in MDR, XDR, and PDR strains were higher than those in antibiotic-sensitive strains. There was also a strong correlation between the regulation of VFs and antibiotic resistance profiles. Moreover, up-regulation of *RNAIII* and *sarA* genes in MRSA strains was observed. These results reflect those of numerous studies that found the overexpression of *RNAIII and sarA* genes in the MRSA strains compared to the MSSA strains. They also found that the *PII* and *PIII* in MRSA strains play an essential role in pathogenicity [13, 24, 25]. The fold change of the *SaeRS, RNAIII,* and *sarA* genes in some *SCCmec* positive isolates showed the highest value in line with the discussed studies. More precisely, our results showed that down-regulation of *RNAII* gene in some *S. aureus* isolates; however, PIII and *sarA* decreased expressions of regulated genes was less than other genes.

In the present study, we found that VF genes were more abundant in MRSA strains than MSSA strains. Further, some isolates carrying all or many VF genes showed high expression of *mecR, mecI, mecA,* and *blaZ* genes. Some researchers from the USA [26], Korea [27], and United Kingdom [28] indicated a strong correlation between VFs and up-regulation of *mecR* and *mecI* genes. They also showed that some VFs of *S. aureus,* such as Panton-Valentine, must play essential roles in distributing SCCmec cassettes. Our studies also provide novel information on how *S. aureus* potentially uses a unique regulatory system to control the expression of its VFs.

In the present study, *mecR, mecI, mecA,* and *blaZ* had a variable expression in toxin and hemolysin-producing isolates. Also, down-regulation of *mecR* and *mecA* was observed in hemolysin non-producing isolates. In non-toxigenic and antibiotic-sensitive strains, *mecI* may play a direct role in the overexpression of β-lactamase regulatory genes. Regarding other reports, the complex role of *mecRI* in the pathogenicity of MRSA strains can be concluded. They also confirmed that the cooperation of VFs and regulating β-lactamase genes also play a critical role in increasing the pathogenicity of *S. aureus* isolates [15, 29–31].

In some research, expression of virulence factors regulatory has been reported to be modulated in the presence of inhibitory concentrations of antibiotics [27]. Our findings are in line with these data. These data suggest the increased level of RNAIII in strains lacking *mecRI* is due to an increase in SarA-mediated transcription of RNAIII. Further, these data confirm a direct linkage between central methicillin expressions and three major virulence regulators (*SarA*, *SaeRS*, and *RNAIII*) in *S. aureus*.

Our results showed a significant relationship between increasing the frequency of antibiotic-resistant strains and *agr* two-component system expression. Some studies have linked reduction in *agr* function with antibiotic tolerance, reduction in the antibacterial activity, and the development of the MRSA phenotype. These changes being described in association with agr type II in the Lee et al. study [32].

A study also linked agr group II polymorphism with poor responses to vancomycin therapy for patients with MRSA infections [19]. In several in vitro studies, the reduction of the *agr* function has been shown to favor the development of antibiotic resistance. It may confer a potential advantage in a hospital setting [12]. Delta-hemolysin is a virulence factor regulated by *agr*: encoded by hld within the *agr* locus; it is a protein derived from the translation of RNAIII, the effector molecule of *agr*. Mutations in *agr* are associated with strongly impaired virulence in various infections with enhanced biofilm formation. Our findings are in line with these data.

In the current study, β-lactam regulatory genes were investigated in this regard and found to induce transcriptional up-regulation of *SaeRS*. Additionally, the *SaeRS* is vital in regulating β-lactamase genes in *S. aureus* infection, and the inactivation of *SaeRS* decreases virulence and resistance. The results would therefore side with the findings of Rapun-Araiz et al. [33]. They confirmed that the sae system regulates the expression of many virulence factors involved in bacterial adhesion, toxicity, and immune evasion.

Also, *SaeRS* two-component system activity is linked with the synthesis of virulence factors and virulence factor regulators, including RNAIII, part of the agr quorum-sensing system. However, it should be noted that in all these cases, the formation of biofilm is one of the most critical factors in increasing antibiotic resistance. In BSI, the *SaeRS* system was activated by the presence of human neutrophil peptides, calprotectin, H2O2, an acidic pH, and sub-inhibitory concentrations of β-lactam antibiotics [33, 34].

According to our observation in BSI isolates, dangerous isolates like ST295, ST30, and ST230 had a high expression level of virulence and β-lactamase regulatory genes. These STs have been frequently reported in BS infections, and increased antibiotic resistance levels have also been seen in this ST. However, ST15 and ST1 were identified as dangerous strains in South Korea reports [3] and Malaysia [35]. In this study, among 91 isolates, 64 unique STs were detected, and the ST30, ST5, and ST22 were the most common STs in *S. aureus* isolates. Moreover, the remaining six isolates were found to belong to ST30. Other reports described a collection of *S. aureus* isolates that commonly belong to ST30 and ST22 [36, 37]. The NBJ tree of the ST30 gene included six isolates into one central cluster.

Regarding virulence genes, *edin* genes were detected in a single isolate of CC30. Several studies performed in different countries have been reported this toxin in other MRSA genetic backgrounds such as CC30 and C22 [15, 38]. This demonstrated consistency with the previous studies. CC30 and CC22 were the only clones that were positive for exfoliative toxins *etA*, *etB* genes. These toxins are responsible for staphylococcal scalded skin syndrome [38, 39]. The prevalence of these genes might be due to the specific geographic region.

We hope to develop a more vital understanding of the virulence in MRSA and MSSA strains in the future. This knowledge may also help clarify the role of β-lactamase enzymes in the pathogenicity and VFs gene expression of *S. aureus*. Therefore, This data could also help investigate the pathogenicity differences between the MRSA and MSSA strains in BSI.

However, the two main limitations of the present study are the lack of biofilm operons and σ factor. Two-component regulatory systems play a critical role in quorum-sensing and biofilm formation. Biofilm formation can also lead to antibiotic resistance in various bacteria. Also, the σ factor plays an essential role in two-component regulatory systems. On the other hand, agr-dependent and SaeRS-dependent systems are affected by the σ factor. Although the role of σ factor in antibiotic resistance is unclear, it is involved in pathogenicity.

## Conclusions

We found a different activity of virulence and β-lactamase regulatory gene in MSSA and MRSA strains. A low expression of *SaeRS*, *RNAIII,* and *sarA* genes was observed in MSSA strains; however, virulence regulatory genes increased MRSA strains’ virulence activity. However, the strong correlation between virulence and β-lactamase regulatory gene indicated that MRSA invasion could be reduced by suppressing any of these regulators. *SCCmec* cassette also plays a vital role in the pathogenicity and activity of PII and PIII promoters*. S. aureus* elaborates on many virulence factors that allow the organism to establish and spread infections under various conditions. The regulation of these virulence factors is intimately linked with antibiotic resistance by antibiotic-resistant regulators. The close linkage of the two-component system and virulence determinant can be exploited to modify the temporal pattern of virulence factor synthesis and attenuate virulence. In addition, increasing two-component system activity could potentially increase the susceptibility of *S. aureus* to bactericidal antibiotics in BSI. The expression of *SaeRS* and alternative *agr* locus and virulence promotors were demonstrated to be inducible by sub-inhibitory concentrations of methicillin and may thus be involved in this process. Further, the two-component system *SaeRS* was shown to play a crucial role in resistance against β-lactam antibiotics. Thus, understanding the risk factors associated with BSI can lead to preventive interventions to reduce complications, including mortality.

## Methods

### Study design, collection, and identification of isolates

Between January 2019 and February 2020 in Hamadan, 639 samples were collected from BSI isolates. These isolates were collected from teaching hospitals’ microbiology laboratories in Hamadan city from Iran. All blood samples which were sent to the diagnostic microbiology laboratory were included in the study. After considering the Inclusion and Exclusion criteria, blood samples are used for culture and further biochemical investigations. Samples are inoculated in Blood Agar (Merck, Germany). Around 91 clinical isolates of *S. aureus* from BSI, which grew beta-hemolytic golden-yellow colonies on 5% sheep blood agar, were processed.

### Antimicrobial susceptibility testing

The disk diffusion method (DDM) and the Clinical & Laboratory Standards Institute (CLSI) guidelines were used for the screening of antibiotic susceptibility profiles [40]. The test was performed on Muller Hinton agar (Merck, Germany). For quality control, *S. aureus* ATCC 25923 and *S. aureus* ATCC 43300 were used.

### DNA extraction and detection of VFs genes

DNA to be used as a PCR template was extracted from *S. aureus* isolates using a simple boiling method. Briefly, *S. aureus* isolates were grown overnight on LB agar. A single bacterial colony was re-suspended in 1000 μl sterile water and centrifuged for 10 mins at 13000 rpm after vortexed. The supernatant was removed, and the pellet was re-suspended in 200 μL of 10 mM EDTA (pH 8) and vortexed, after incubated at 100 °C for 30 min. After boiling, 100 μL of 10 mM Tris-HC (pH 8) was added and centrifuged for 5 mins at 13000 rpm after vortexed. The resulting supernatant was used as a template for PCR [41]. Finally, by Nanodrop (Hangzhou, China), the DNA concentration was measured.

According to Table 3, the VFs, antibiotic resistance, and SCCmec genes were amplified. The Eppendorf PCR machine (Eppendorf, Mastercycler® 5332, Germany) was used for amplifying genes. The 25 μl reaction mixture contained 12.5 μl of master mix (Ready Mix TM-Taq PCR Reaction Mix, Ampliqon, Denmark), 0.5 μM concentration of each primer, 1 μl of the genomic DNA template, and 11.5 μL of molecular biology grade water. In each round of amplification, sterile water was used as a negative control.

**Table 3:** Primers used for identification of virulence factors genes, SCCmec types, real-time PCR of β-lactamase and virulence regulatory genes BSI collection of *S. aureus*

Genes	Primers	Ref
*tst*	F: TTCACTATTTGTAAAAGTGTCAGACCCACT R: TACTAATGAATTTTTTTATCGTAAGCCCTT	[12]
*etA*	F: ACTGTAGGAGCTAGTGCATTTGT R: TGGATACTTTTGTCTATCTTTTTCATCAAC	[12]
*etB*	F: CAGATAAAGAGCTTTATACACACATTAC R: AGTGAACTTATCTTTCTATTGAAAAACACTC	[12]
*lukS-PVL*	F: ATCATTAGGTAAAATGTCTGGACATGATCCA R: GCATCAASTGTATTGGATAGCAAAAGC	[12]
*LukE-LukD*	F: TGAAAAAGGTTCAAAGTTGATACGAG R: TGTATTCGATAGCAAAAGCAGTGCA	[12]
*lukM*	F: TGGATGTTACCTATGCAACCTAC R: GTTCGTTTCCATATAATGAATCACTAC	[12]
*edinA*	F: GAAGTATCTAATACTTCTTTAGCAGC R: TCATTTGACAATTCTACACTTCCAAC	[12]
*hla*	F: CTGATTACTATCCAAGAAATTCGATTG R: CTTTCCAGCCTACTTTTTTATCAGT	[12]
*hlb*	F: GTGCACTTACTGACAATAGTGC R: GTTGATGAGTAGCTACCTTCAGT	[12]
*hld*	F: AAGAATTTTTATCTTAATTAAGGAAGGAGTG R: TTAGTGAATTTGTTCACTGTGTCGA	[12]
*hlg*	F: GTCAYAGAGTCCATAATGCATTTAA R: CACCAAATGTATAGCCTAAAGTG	[12]
*SCCmecI*	F: GCTTTAAAGAGTGTCGTTACAGG R: GTTCTCTCATAGTATGACGTCC	[4]
*SCCmecII*	F: CGTTGAAGATGATGAAGCG R: CGAAATCAATGGTTAATGGACC	[4]
*SCCmecIII*	F: CCATATTGTGTACGATGCG R: CCTTAGTTGTCGTAACAGATCG	[4]
*SCCmecIV*	F: GCCTTATTCGAAGAAACCG R: CTACTCTTCTGAAAAGCGTCG	[4]
*SCCmecV*	F: GAACATTGTTACTTAAATGAGCG R: TGAAAGTTGTACCCTTGACACC	[4]
*mecRI*	F: TGGTATTTGGTTTAGTGAA R: GATTAGGTTTAGGCATTGA	[32]
*mecI*	F: AATGGCGAAAAAGCACAACA R: GACTTGATTGTTTCCTCTGTT	[32]
*blaz*	F: AAGAGATTTGCCTATGCTTC R: GCTTGACCACTTTTATCAGC	[33]
*mecA*	F: AAAGAACCTCTGCTCAACAAGT R: TGTTATTTAACCCAATCATTGCTGTT	[33]
*RNAII*	F: TATGAATAAATGCGCTGATGATATACCACG R: TTTTAAAGTTGATAGACCTAAACCACGACC	[34]
*RNAIII*	F: GCCATCCCAACTTAATAACCA R: TGTTGTTTACGATAGCTTACATGC	[34]
*sarA*	F: TCTTGTTAATGCACAACAACGTAA R: TCTTGTTAATGCACAACAACGTAA	[34]
*agrA*	F: ATGCACATGGTGCACATGC R: GTCACAAGTACTATAAGCTGCGAT	[11]
*agrB*	F: ATGCACATGGTGCACATGC R: GTATTACTAATTGAAAAGTGCCATAGC	[11]
*agrC*	F: ATGCACATGGTGCACATGC R: CTGTTGAAAAAGTCAACTAAAAGCTC	[11]
*agrC*	F: ATGCACATGGTGCACATGC R: CGATAATGCCGTAATACCCG	[11]
*seaRS*	F: GCTCATGCTTCTGAGCAAGA R: CTAATACGACTCACTATAGGGAGA	[14]
*gmk*	F: TCGTTTTATCAGGACCATCTGGAGTAGGTA R: CATCTTTAATTAAAGCTTCAAACGCATCCC	[34]

### RNA extraction, synthesis of cDNA and qRT- PCR assay

RNA extraction and cDNA synthesis were performed by GeneAll mini kit (GeneAll Biotechnology, Korea) [42]. Relative quantification was carried out using the Cycle Threshold (CT) comparative method. qRT-PCR reactions were performed in 96-well microplates (ABI-Step One-Plus) using the ABI-Step One-Plus Real-time System, ABI, USA. Components used for each reaction in q-PCR were 1 μl aliquot of the first-strand cDNA in a final volume of 20 μl; containing 10 pM of specific primers (forward and reverse) was used. PCR was carried out using 4 μl 2x FIREPol Master Mix RTL MgCl2 Master Mix (Solis BioDyne, Tartu, Estonia), primer forward and reverse both were 0.5 μl, template cDNA 2 μl, and RNase free water 13 μl to final volume 20 μl. The PCR protocol was designed for 40 cycles, and a melting-curve analysis (65 °C to 95 °C, fluorescence read every 0.3 °C) was performed to check the specificity of the amplification. Relative quantification was achieved using the CT comparative method [43]. For quality control, *S. aureus* ATCC 25923 and *S. aureus* ATCC 43300 were used in the study.

### Generating heat maps of expression data

For the drawing of the heatmap, the One Matrix CIM online package (https://discover.nci.nih.gov/cimminer/home.do) was used. To obtain a preliminary overview of the gene expression data, all log_2_-fold changes generated by Bioconductor programmer DESeq2 (v1.14.1) for the test conditions compared to the control were input into the heatmap.2 regardless of the adjusted *p*-value.

### Multi-locus sequence typing of isolates

Multi-locus sequence typing (MLST) for *S. aureus* was performed, according to Tahmasebi et al. [15]. MLST of *S. aureus* was performed based on the sequences of seven housekeeping genes *arcC*, *aroE*, *glpF*, *gmk*, *pta*, *tpi*, and *yqiL* (https://pubmlst.org/organisms/staphylococcus-aureus). MLST database (https://pubmlst.org/bigsdb?db=pubmlst_saureus_seqdef&page=profiles&scheme_id=1) also used for the determination of STs.

### Statistical analysis

All statistical analyses were performed using the Prism GraphPad, version 8.0 (GraphPad Software, Inc., CA, US). The relationship between categorical variables was compared using the χ^2^ test. Further analysis was performed in DataAssist (Applied Biosystems, CA, USA). The *p*-value was calculated based on a two-sample, two-tailed Student’s t-test for the calculated. Fold change (relative to the epilepsy group) and a p-value were generated on a two-sample, two-tailed Student’s t-test. One Matrix CIM online package (https://discover.nci.nih.gov/cimminer/home.do) was used for drawing the heatmap and hierarchical clustering. Expression analysis data were taken in three replication and given as mean value ± SE.

## Supplementary Information

**Additional file 1.**

## Data Availability

The datasets used and/or analyzed during the current study are available from the corresponding author on reasonable request.

## Abbreviations

VFs: Virulence factor
MRSA: Methicillin-resistant *Staphylococcus aureus*
*SCCmec*: Staphylococcal cassette chromosome *mec*
HA-MRSA: Associated with healthcare-associated MRSA
TSST-1: Toxic shock syndrome toxin 1
MDR: Multidrug-resistant
XDR: Extensively drug-resistant
PDR: Pandrug-resistant
MLST: Multi-locus sequence typing
ST: Sequence type
CT: Cycle threshold
