# Supplementary material for: Regulation of virulence and β-lactamase gene expression in Staphylococcus aureus isolates: cooperation of two-component systems in bloodstream superbugs
Source: BMC Microbiol. 2021 Jun 25;21:192. doi: 10.1186/s12866-021-02257-4 (PMC8228909; doi:10.1186/s12866-021-02257-4)
Supplement: Supplementary file 1 — Additional file 1. [file 12866_2021_2257_MOESM1_ESM.docx]

Dear Bryan Bulanadi
Editorial Support Team
BMC

Thanks for your message.

I really appreciated for taking time.

About your query:

1. Please note that the title in your manuscript file differs from the title entered in the submission system - please correct so they are consistent with each other.

Response: I checked the title and corrected it

2. Please note that BMC mandates data deposition for the following:

• Protein sequences
• DNA and RNA sequences
• DNA and RNA sequencing data
• Genetic polymorphisms
• Linked genotype and phenotype data
• Macromolecular structure
• Microarray data (must be MIAME compliant)
• Crystallographic data for small molecules

As such, we ask that you please provide the relevant accession numbers if the data has been deposited into a data bank or the appropriate web links if the data has been uploaded into a repository. If the data has not yet been deposited, please do so and provide the relevant information needed to access it in the ‘Availability of data and materials’ section in your manuscript. Please ensure all data is already released and that any accession numbers/web links are in the ‘Availability of Data and Materials’ in their final form, and that all links and accessions allow access to public data.

If applicable, your data availability statement may include the following: “The datasets generated and/or analyzed during the current study are available in the [NAME] repository, [PERSISTENT WEB LINK OR ACCESSION NUMBER TO DATASETS]”.

Response: as you know in this study we didn’t introduce any new gene which need to submit to the gene bank for taking accession number, we just detected the existence gene which introduced by another researchers and we referenced in table 1. As well about MLST you could see the explanation in below

Multi-locus sequence typing (MLST) is a method used for epidemiological studies. Approximately 400-450 bp of seven genes (housekeeping genes) necessary for the maintenance of the bacteria are determined. For each isolate, the alleles of the seven genes represent an allelic profile. This allelic profile can be compared with a publicly available MLST database containing all published alleles resulting in the assigning of a sequence type (ST). By using one commonly recognized MLST scheme, the result can be compared to or analyzed with other isolates examined elsewhere in the world. Every unique sequence is given a distinct allele number and each unique combination of alleles is assigned a distinct sequence type (ST) number. The results are highly reproducible and the data are archived in the form of the Web based databases in order to enable easy exchange of the information. MLST can be automated for high-throughput.

However, in the present study, the MLST scheme published by pubMLST.org was used, with a few additional primers, which involve the genes *arc, arcC, aroE, glpF, gmk, tpi, pta* and *yqi*. The clonality of the results was analyzed using eBURSTv3 (http://eburst.mlst.net/), a web-based program that analyses a collection of samples regarding similarity in the allelic profiles and based on the similarities divide the STs into clonal complexes. Due to the above description, no new gene has been introduced and identified in the present study. In other words, we examined MLST typing in different strains. In strain typing, gene registration or accessions number is not important. Identifying STs and CCs is the most important part of MLST typing that was done accurately in the present study. However, raw file sequences based on MLST typing are available.
